# Recurrent multifocal diabetic myonecrosis: a cause of severe extremity pain in a diabetic patient

**DOI:** 10.1308/003588413X13511609956651

**Published:** 2013-01

**Authors:** MJ Welck, P Vetpillai, V Balaji, R Jennings, WD Goodier

**Affiliations:** ^1^Barts and The London NHS Trust,UK; ^2^Basildon and Thurrock University Hospitals NHS Foundation Trust,UK

**Keywords:** Recurrent, Diabetic myonecrosis, Treatment, Diagnosis

## Abstract

We detail the unusual occurrence of successive episodes of diabetic myonecrosis in both lower and upper limbs in a young woman with diabetic end organ dysfunction. We highlight the importance of recognising this as a differential diagnosis for acute limb pain in diabetic patients and advocate conservative measures for successful treatment and return to function.

## Case history

A 26-year-old black woman with type 1 diabetes mellitus was referred to the orthopaedics team with severe anteromedial right thigh pain. Over the preceding four weeks, a tender mass had formed in her anteromedial thigh. The patient had a 16-year history of poorly controlled type 1 diabetes mellitus with end stage diabetic nephropathy requiring regular haemodialysis. She had had a similar episode in the contralateral thigh six months earlier.

On examination she was unable to bear weight. A 10cm × 15cm firm, fixed mass was present in her right anteromedial thigh. The range of motion of the ipsilateral hip and knee were within normal limits but provoked severe pain in the thigh. The calf was supple and non-tender to palpation. Both limbs were warm and well perfused. There was no history of trauma and the area was not the site of local insulin injection. There were no systemic symptoms.

Initial investigations revealed a normal white blood cell count (10.0 × 10^9^/l), raised C-reactive protein (CRP) levels (249mg/l), raised creatine kinase (CK) levels (661iu/l, normal: 25–150iu/l) and a raised erythrocyte sedimentation rate (ESR) (119mm/hr, normal: 0–25mm/hr). Intracompartmental pressure was normal (10mmHg anteriorly, 9mmHg medially and 10mmHg posteriorly; diastolic blood pressure 94mmHg).

**Figure 1:**
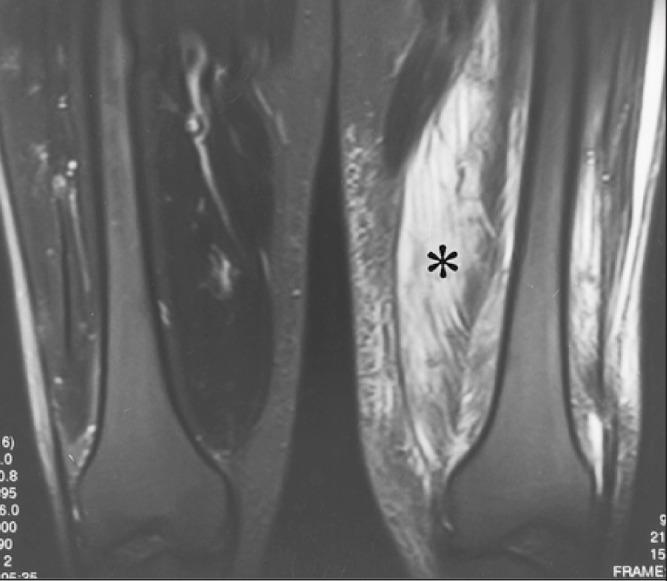
Magnetic resonance imaging following intravenous gadolinium revealing relative areas of serpiginous non-enhancement in the left vastus medialis and vastus intermedius indicative of necrosis

No bone abnormality was demonstrated on plain x-rays or computed tomography of the thigh. Ultrasonography demonstrated extensive subcutaneous oedema, enlargement of the medial thigh musculature with indistinct fibres and loss of the appearance of the fascia. Magnetic resonance imaging (MRI) revealed extensive muscle oedema with increased signal intensity in all the quadriceps muscle and some of the adductors with central necrosis (Fig 1). There was relative sparing of the hamstrings.

The patient was admitted for bed rest, analgesia and glycaemic control. She was discharged after 14 days following significant improvement in her pain and blood parameters (CRP: 38mg/l, CK: 102iu/l). However, she was readmitted 11 days later with identical pain inferolateral to the previous site. Blood investigations revealed a raised CRP of 148mg/l, CK of 435iu/l and ESR of 119mm/hr. MRI confirmed a second successive episode of myonecrosis in the quadriceps. Three weeks into her second admission she developed an exquisitely painful mass in the anterolateral aspect of her right arm. MRI demonstrated patchy areas of abnormal enhancement in the biceps muscle suggestive of myonecrosis. The pain and swelling resolved gradually with conservative treatment.

## Discussion

Diabetic myonecrosis is a rare complication of diabetes. Only one case in the forearm musculature has been reported previously.^1^ Ours is the first report to describe rapidly recurrent episodes of diabetic myonecrosis involving the musculature of both lower and upper limbs.

There is a predilection for women and there is a greater incidence in patients with type 1 diabetes.^2^ As with our case, patients usually have established multiple complications of diabetes such as nephropathy (71%), retinopathy (56%) and neuropathy (54%).^3^ Diabetic myonecrosis is most common in the quadriceps (87%) but can rarely affect the hip abductors and hamstrings.^3^ The pain is at rest and muscle function appears to be normal. Eighty per cent of previous cases describe an abrupt onset of pain in the affected muscle accompanied by localised swelling in the absence of trauma or fever with subsequent partial resolution and appearance of a painful palpable mass.^1–3^

The precise pathogenesis is disputed but it is likely to be related to diabetic microangiopathy.^1^ Even though Kapur *et al* reported that leucocytosis, elevated CK levels and an elevated ESR are found in 35%, 45% and 75% of cases respectively, routine laboratory investigations are not helpful in diagnosis.^2,5^

The most valuable diagnostic tool is T2 weighted, inversion recovery and gadolinium enhanced MRI. Ultrasonography may help differentiate diabetic myonecrosis from a necrotic mass or abscess. Muscle biopsy can confirm the diagnosis but may lead to potential healing complications. Analgesia, bed rest and tight glycaemic control form the mainstay of treatment.^1–3^ The short-term prognosis of diabetic myonecrosis is good although recurrence has been reported in up to a half of cases.^1^

## Conclusions

The surgeon must have a high index of suspicion of diabetic myonecrosis in a diabetic patient with end organ complications who presents with an acutely painful limb in the absence of trauma. T2 weighted MRI is the investigation of choice and the treatment is conservative.
